# Psychometric evaluation of the Chinese version of the older adult lifestyle scale: a translation and validation study

**DOI:** 10.3389/fpubh.2025.1539685

**Published:** 2025-04-28

**Authors:** Mengmeng Bi, Huifeng Yan, Lin Liu, Jian Zhu, Wenguang Xie, Li Zhang

**Affiliations:** ^1^Comprehensive Intervention Room, The Second Affiliated Hospital of Nanchang University, Nanchang, China; ^2^Imaging Center, The Second Affiliated Hospital of Nanchang University, Nanchang, China; ^3^Department of Otolaryngology, Head and Neck Surgery, the Second Affiliated Hospital of Nanchang University, Nanchang, China; ^4^The 2nd Affiliated Hospital, Jiangxi Medical College, Nanchang University, Nanchang, China

**Keywords:** older people, lifestyle, psychometric properties, factor analysis, scale

## Abstract

**Objective:**

The objective of this study was to translate the Older Adult Lifestyle Scale (OALS) from English to Chinese and to assess the psychometric characteristics of the Chinese version of the OALS.

**Methods:**

In this study, the Brislin two-way translation method was employed to translate the OALS into Chinese. Between June 2023 and February 2024, a total of 393 older adults were recruited from the provinces of Jiangxi, Anhui, Guizhou, and Heilongjiang in China using a convenience sampling method to assess the psychometric characteristics of the Chinese version of the OALS. The reliability of the scale was evaluated through split-half reliability, test–retest reliability, and internal consistency. The validity of the scale was assessed using the Delphi expert correspondence method and factor analysis.

**Results:**

The Chinese version of the OALS comprises four dimensions and 19 items, demonstrating a Cronbach’s α coefficient of 0.824. The Cronbach’s α coefficients for the four dimensions range from 0.867 to 0.951, with a split-half reliability of 0.792 and a retest reliability score of 0.964. In this study, I-CVI ranges from 0.857 to 1.000, while S-CVI values are 0.955. Exploratory factor analysis indicated a KMO value of 0.846 and a Bartlett’s sphericity test χ^2^value of 3397.370 (*p* < 0.001). Four common factors were extracted: preventive behavior, food/diet, physical activity, and quality of relationships. The cumulative variance contribution rate was 76.682%, and the factor loadings for all items were satisfactory. The results of the confirmatory factor analysis revealed the following fit indices: CMIN/DF = 1.446, RMSEA = 0.053, CFI = 0.979, GFI = 0.901, TLI = 0.976, and IFI = 0.980, indicating that all fitting indices were satisfactory.

**Conclusion:**

The Chinese version of the OALS demonstrates robust and reliable psychometric properties and serves as an effective instrument for assessing the lifestyle of older adult individuals. The utilization of this scale can be beneficial for medical professionals and government agencies, as it encompasses preventive behaviors, dietary habits, physical activity, and the quality of interpersonal relationships. These factors collectively provide a foundation for developing lifestyle intervention programs tailored for the older adult population.

## 1 Introduction

With the intensification of the global aging phenomenon, the health management and nursing of the older adult has become an increasingly important issue in the field of public health. The World Health Organization predicts that by 2050, there will be 2.1 billion individuals over the age of 60 and 426 million over the age of 80 worldwide, with 80% of the older adult residing in low- and middle-income countries (1). China is among these countries. According to data from China’s seventh population census, as of midnight on November 1, 2020, the older adult population aged 60 and older in China is 264,018,766, accounting for 18.70% of the total population (2). Currently, China is experiencing a significant aging phase, characterized by an accelerated pace of population aging. This demographic shift, coupled with a shortage of medical service resources, a high prevalence of chronic diseases, and rising medical costs, poses increasingly severe challenges to the health management of the older adult (3).

A lifestyle is characterized by a series of behaviors that are consistently repeated in an individual’s daily life (4). The lifestyle of the older adult population is closely linked to their health; in fact, a positive lifestyle not only effectively prevents chronic diseases but also enhances the quality of life for older adults. Research indicates that an active lifestyle is positively correlated with mental health, life satisfaction, physical well-being, and longevity among the older adult, while it is negatively correlated with cognitive decline (5–7). Investigations into the dietary habits and physical exercise of older adults reveal that a healthy diet combined with moderate exercise can significantly reduce the risk of chronic diseases and delay functional decline (8, 9). Foreign studies have demonstrated that a healthy lifestyle can mitigate cognitive decline in the aging population. Furthermore, the risk of dementia can be reduced through adherence to a balanced diet and active social participation (10, 11). Furthermore, a healthy lifestyle is closely associated with alleviating the economic burden on the older adult (12). Thus, assessing and improving the lifestyles of older adults, as well as encouraging them to adopt healthier habits, has become a crucial objective for public health interventions.

In China, the lifestyle of the older adult is shaped by cultural, social, economic, and personal factors, highlighting an urgent need for the establishment of local assessment tools (3, 13). Currently, however, there is no effective instrument available for evaluating the lifestyles of the older adult in China. In 2023, Professor Ferreira et al. from Brazil developed the Older Adult Lifestyle Scale (OALS) using a mixed-methods research approach (4). This scale provides a comprehensive assessment of older adult lifestyles across four dimensions: Preventive Behavior, Diet, Physical Activity, and Quality of Relationships. It is well-designed, user-friendly, and has demonstrated good reliability and validity among the older adult population in Brazil, making it suitable for broader application. Nevertheless, this scale has yet to be utilized in other countries, including China, and there are no existing reports on relevant reliability and validity studies.

The aim of this study was to translate the English version of the OALS into Chinese and to evaluate its psychometric properties within the older adult population of China. This endeavor seeks to provide a reliable tool for assessing the lifestyles of older individuals in China. The results of this study will offer a scientific basis for the formulation of public health policies and will serve as a reference for implementing measures aimed at promoting healthy lifestyle among the older adult.

## 2 Methods

### 2.1 Design and participants

The multicenter cross-sectional study was conducted between June 2023 and February 2024 across four Chinese provinces: Jiangxi, Anhui, Guizhou, and Heilongjiang. A total of 393 older individuals were recruited from these provinces using a convenience sampling method. The study took place in a community street office, where researchers conducted face-to-face interviews with respondents. Researchers underwent standardized training to ensure consistent delivery of instructions. Interview protocols included scripted introductions and neutral prompts to minimize bias. In this study, a power analysis was adopted for sample size. According to the sample size requirements for quantitative research, each project should include at least 10 participants (14). To ensure the authenticity and accuracy of the research results, it was determined that a minimum of 20 older adult individuals should participate in each item of the study. With 19 items in the questionnaire, this necessitated the recruitment of 380 older adult participants. However, to account for potential non-responses or invalid questionnaires, a larger sample size was deemed necessary, resulting in the final recruitment of 393 older adult individuals for this study (15). The inclusion criteria for participants were: aged over 60 years, conscious, able to communicate normally, and voluntarily participating in the study.

### 2.2 Measures

#### 2.2.1 General demographic characteristics of the older adult questionnaire

After a thorough literature review and group discussion, the research team designed a general demographic characteristics questionnaire for the older adult suitable for this study. The questionnaire included seven questions: gender, age, marital status, education level, number of basic diseases, monthly family income and living conditions.

#### 2.2.2 Burnout syndrome assessment scale

The Older Adult Lifestyle Scale (OALS) was developed by Ferreira et al. (4). The scale includes four dimensions: Preventive Behavior、Food/diet, Physical Activity and Quality of Relationships, and consists of 19 items. Each item was evaluated using a 5-point Likert scale, with scores ranging from 1 to 5 points (1 = never, 2 = almost never, 3 = occasionally, 4 = frequently, 5 = always). Scores ranged from 19 to 95, with higher scores indicating a healthier lifestyle.

### 2.3 Procedures

#### 2.3.1 Scale translation procedure

After contacting Professor Ferreira via email and obtaining authorization for the scale, the scale was translated into Chinese through a process of translation and cross-cultural adaptation. In this study, the Brislin two-way translation method (16) was employed to facilitate the translation of the scale into Chinese. Initially, the OALS was translated into Chinese by two Chinese professors specializing in English. The research team then discussed and reached consensus on sections that significantly deviated from the original content. Subsequently, two native English-speaking professors independently translated the scale back into English without referring to the original version. To ensure the translated scale aligned with Chinese linguistic norms, two nursing experts, two gerontology experts, and two psychology experts were invited to evaluate it and provide suggestions for modification. Ultimately, the Chinese version of the OALS was finalized by incorporating the experts’ recommendations. Eleven older adult individuals were selected for a pre-survey to assess the clarity and comprehensibility of the scale items. The older adult participants reported that the scale was easy to understand, with no ambiguity, and it took approximately 4–5 min to complete.

#### 2.3.2 Data collection procedure

Following the training, the research team employed convenience sampling to travel to cities across four provinces for participant recruitment. Researchers adhered to uniform guidelines to articulate the purpose and significance of the study to the older adults involved. Each participant was required to sign an informed consent form. A total of 420 older adult individuals were recruited for the study; however, 22 declined to participate due to prior commitments, leaving 398 older adult individuals who were brought to the community street office for face-to-face interviews to complete the questionnaire. All questionnaires were administered anonymously. Ultimately, 5 invalid questionnaires were excluded, resulting in 393 valid responses and an effective recovery rate of 98.74%. Additionally, 33 older adult individuals were assigned numbers and invited to complete a questionnaire 2 weeks later to assess the reliability of the scale through retesting.

#### 2.3.3 Data analysis procedure

In this study, SPSS 25.0 software and AMOS 24.0 software were used for statistical analysis. Descriptive statistical methods were used to describe the characteristics of the participants. *p* < 0.05 was considered to be statistically significant.

##### 2.3.3.1 Items analysis

The critical ratio method (CR), correlation coefficient method, and homogeneity test method were employed to analyze the items. The critical ratio method reflects the degree of differentiation among scale items by calculating the total score of each questionnaire and ranking them from low to high. The lowest 27% (low group) and the highest 27% (high group) are then subjected to a two-independent sample T-test. It is generally accepted that a critical ratio of each item ≥3 and *p* < 0.05 indicates good differentiation of the item (17). The Pearson correlation test was utilized to assess the correlation between the score of each item and the total score of the scale, thereby evaluating the homogeneity of the items. A correlation coefficient of ≥0.4 between the score of each item and the total score of the scale is typically regarded as indicative of appropriate homogeneity (18). Furthermore, an analysis of Cronbach’s α coefficient after the removal of an item suggests that if the Cronbach’s α coefficient increases upon deletion, this implies that the attribute measured by the item differs from those of the other items, indicating that the item may warrant consideration for deletion (19).

##### 2.3.3.2 Reliability analysis

Reliability is an index that measures the accuracy and consistency of a measuring instrument’s responses to the results obtained, reflecting the true degree of the characteristics being measured (17, 20). In this study, we employed Cronbach’s α coefficient, split-half reliability, and test–retest reliability to evaluate the internal consistency of the Chinese version of the scale. We assert that a Cronbach’s α coefficient, split-half reliability, and test–retest reliability all equal to or greater than 0.70 indicate that the reliability of the Chinese version of the scale is satisfactory (21, 22).

##### 2.3.3.3 Validity analysis

This study analyzes the validity of the scale from two perspectives: content validity analysis and structural validity analysis. Content validity refers to the degree to which the items of the research tool accurately reflect the content being measured. In this study, three nursing specialists and four gerontologists were invited to evaluate the content validity of the questionnaire using the Delphi expert correspondence method. A 4-point Likert scale was employed to collect feedback from the experts, with ratings ranging from 1 (not relevant) to 4 (highly relevant). The content validity index (I-CVI) at the item level and the scale-level content validity index (S-CVI) were calculated successively based on the expert ratings. The I-CVI is determined by the formula: Number of experts with a score of 3 or above divided by the total number of experts, with a threshold of greater than 0.78 required for validity (15, 23). The S-CVI is calculated as the average of the I-CVI scores for the 19 items. A S-CVI of 0.90 or higher indicates good content validity for the translated scale (15, 23).

In this study, exploratory factor analysis (EFA) and confirmatory factor analysis (CFA) were employed to assess the structural validity of the scale. Using SPSS 25.0 software, 393 older adult patients were randomly divided into two groups: one group (*n* = 193) for EFA and the other group (*n* = 200) for CFA. The translated scale is deemed suitable for factor analysis if the Kaiser-Meyer-Olkin (KMO) measure is greater than 0.60 and the Bartlett’s test of sphericity is statistically significant (*p* < 0.05) (24). Amos 24.0 software was utilized for CFA to evaluate the goodness of fit of the model. A well-fitting model is indicated by a Chi-square to degrees of freedom ratio (CMIN/DF) of 3.0 or less, a root mean square error of approximation (RMSEA) of 0.08 or less, and Comparative Fit Index (CFI), Goodness-of-Fit Index (GFI), Tuckey-Lewis Index (TLI), and Incremental Fit Index (IFI) all equal to or greater than 0.90 (25, 26).

### 2.4 Ethical approval

All participants volunteered to participate in the study and signed a written informed consent, all questionnaires were filled out anonymously, and all participants’ information was protected. In addition, this study was approved by the Ethics Committee of the Second Affiliated Hospital of Nanchang University (Approval number: O-Medical Research Lun Review [2023] No. (46)).

## 3 Results

### 3.1 Descriptive statistics

A total of 393 older adults were recruited for the study, comprising 179 men (45.6%) and 214 women (54.4%). Among the participants, 33.3% were aged between 60 and 65 years, 82.7% were married, 34.9% had attained primary school education or below, and 27.2% had a chronic illness. Additionally, 45.3% reported a monthly household income ranging from 3,000 to 5,000 yuan, while 81.2% lived with their families. Further socio-demographic information is presented in Table 1.

**Table 1:** General demographic characteristics of the older adult surveyed (*n* = 393).

Factors	Group	*n*	%
Age	60–65	131	33.3
	66–70	126	32.1
	71–75	61	15.5
	76–80	39	9.9
	>80	36	9.2
Sex	Male	179	45.6
	Female	214	54.4
Marital status	Unmarried	3	0.8
	Married	325	82.7
	Divorced	12	3.0
	Widowed	53	13.5
Education level	Primary and below	137	34.9
	Junior high school	108	27.5
	Technical secondary school	76	19.3
	College or above	72	18.3
Number of underlying diseases	0	92	23.4
	1	107	27.2
	2	105	26.7
	≥3	89	22.7
Monthly household income (Yuan)	≤3,000	93	23.7
	3,000–5,000	178	45.3
	≥5,000	122	31.0
Living condition	Live alone	74	18.8
	Non-solitary	319	81.2

### 3.2 Item analysis

In this study, the critical ratio (CR) values for the 19 items in the scale ranged from 3.134 to 18.235, all exceeding 3.0 with a significance level of *p* < 0.005. These differences were statistically significant, indicating effective differentiation among the items in the scale. The correlation coefficient (r) between each item of the translation scale and the total score ranged from 0.419 to 0.687 (*p* < 0.001), reflecting a moderate correlation between each item and the overall scale. Each of the 19 items was successively deleted, and the reliability of the scale was compared to that of the total scale after the removal of individual items. The results indicated that the Cronbach’s α coefficient ranged from 0.804 to 0.824 upon the deletion of single items, which did not exceed the reliability of the total scale. Consequently, all 19 items of the scale should be retained (Table 2).

**Table 2:** Item analysis for Chinese version of the OALS.

Item	Critical ratio	Correlation coefficient between item and total score	Cronbach’s Alpha if item deleted
Preventive behavior-1	11.219	0.532	0.817
Preventive behavior-2	9.372	0.450	0.822
Preventive behavior-3	8.156	0.419	0.824
Preventive behavior-4	9.225	0.458	0.823
Preventive behavior-5	8.229	0.433	0.822
Food/diet-1	10.113	0.522	0.816
Food/diet-2	12.182	0.598	0.812
Food/diet-3	11.706	0.609	0.810
Food/diet-4	10.834	0.551	0.813
Physical activity-1	3.394	0.427	0.824
Physical activity-2	3.196	0.499	0.824
Physical activity-3	3.170	0.420	0.824
Physical activity-4	3.134	0.495	0.824
Quality of relationships-1	16.119	0.624	0.809
Quality of relationships-2	14.179	0.606	0.809
Quality of relationships-3	18.235	0.687	0.804
Quality of relationships-4	17.170	0.676	0.805
Quality of relationships-5	15.383	0.649	0.806
Quality of relationships-6	17.279	0.683	0.804

### 3.3 Reliability analysis

The Cronbach’s α value for the Chinese version of the lifestyle scale for the older adult was found to be 0.824. The α values for the four dimensions of the scale ranged from 0.867 to 0.951. The split-half reliability of the translated scale was measured at 0.792. After a two-week interval, a sample of 33 older adult individuals was selected for retesting, yielding a retest reliability of 0.964 (Table 3).

**Table 3:** Reliability analysis for Chinese version of the OALS.

The scale and its dimension	Cronbach’s Alpha	Split-half reliability	Test–retest reliability
The OALS	0.824	0.792	0.964
Preventive behavior	0.882		
Food/diet	0.867		
Physical activity	0.947		
Quality of relationships	0.951		

### 3.4 Validity analysis

#### 3.4.1 Content validity analysis

The Content validity analysis was evaluated by three nursing specialists and four gerontologists from China. All experts hold senior titles, ensuring high authority and reliability. The Item-Level Content Validity Index (I-CVI) and the Scale-Level Content Validity Index (S-CVI) were calculated based on the scores provided by the seven experts. In this study, the I-CVI ranges from 0.857 to 1.000, while the S-CVI is 0.955 (Table 4).

**Table 4:** Content validity analysis for Chinese version of the OALS.

Items	Expert1	Expert2	Expert3	Expert4	Expert5	Expert6	Expert7	I-CVI	S-CVI
1	4	4	4	4	4	4	4	1.000	0.955
2	4	4	4	4	4	4	4	1.000	
3	4	4	4	4	4	4	4	1.000	
4	4	4	4	4	3	4	4	1.000	
5	4	4	4	2	3	3	4	0.857	
6	4	4	4	3	3	2	3	0.857	
7	4	4	4	3	3	2	3	0.857	
8	3	4	4	4	4	4	4	1.000	
9	4	4	4	2	4	4	4	0.857	
10	4	4	4	4	4	4	4	1.000	
11	4	4	4	4	4	4	4	1.000	
12	4	4	4	4	3	3	3	1.000	
13	4	4	4	4	3	3	3	1.000	
14	3	3	3	3	3	4	4	1.000	
15	4	4	4	4	4	2	3	0.857	
16	4	4	4	4	4	4	3	1.000	
17	4	4	4	4	4	4	4	1.000	
18	4	4	4	4	2	3	3	0.857	
19	3	3	3	3	4	4	4	1.000	

#### 3.4.2 Exploratory factor analysis

**Figure 1:**
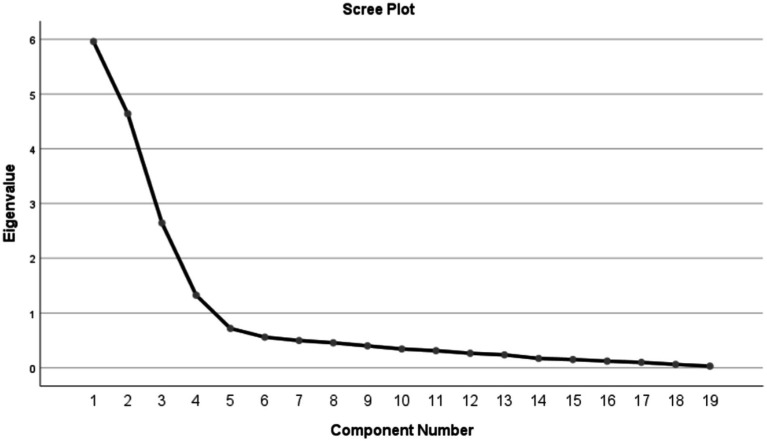
Screen plot of exploratory factor analysis for Chinese version of the OALS.

In this study, the KMO value was 0.846, exceeding the threshold of 0.70, and the Bartlett’s test of sphericity was statistically significant (χ^2^ = 3397.370, *p* < 0.001). These results indicate that the translated lifestyle scale for the older adult is appropriate for factor analysis, and the model demonstrates a good fit. Following the application of Principal Component Analysis (PCA) with maximum variance orthogonal rotation, a total of four factors with eigenvalues greater than 1 were extracted, and these extracted factors were highly consistent with the original scale. The cumulative variance contribution rate was 76.682%, with the factor loadings for each item exceeding 0.4, and no instances of multi-factor loading were observed (Table 5). Furthermore, the scree plot provided additional evidence supporting the existence of the four-factor structure of the scale (Figure 1).

**Table 5:** Factor loadings of exploratory factor analysis for Chinese version of the OALS.

Item	Factor 1	Factor 2	Factor 3	Factor 4
Preventive behavior-1	–	–	0.799	–
Preventive behavior-2	–	–	0.807	–
Preventive behavior-3	–	–	0.832	–
Preventive behavior-4	–	–	0.795	–
Preventive behavior-5	–	–	0.723	–
Food/diet-1	–	–	–	0.740
Food/diet-2	–	–	–	0.794
Food/diet-3	–	–	–	0.813
Food/diet-4	–	–	–	0.746
Physical activity-1	–	0.931	–	–
Physical activity-2	–	0.958	–	–
Physical activity-3	–	0.964	–	–
Physical activity-4	–	0.843	–	–
Quality of relationships-1	0.859	–	–	–
Quality of relationships-2	0.805	–	–	–
Quality of relationships-3	0.906	–	–	–
Quality of relationships-4	0.904	–	–	–
Quality of relationships-5	0.896	–	–	–
Quality of relationships-6	0.879	–	–	–

#### 3.4.3 Confirmatory factor analysis

**Figure 2:**
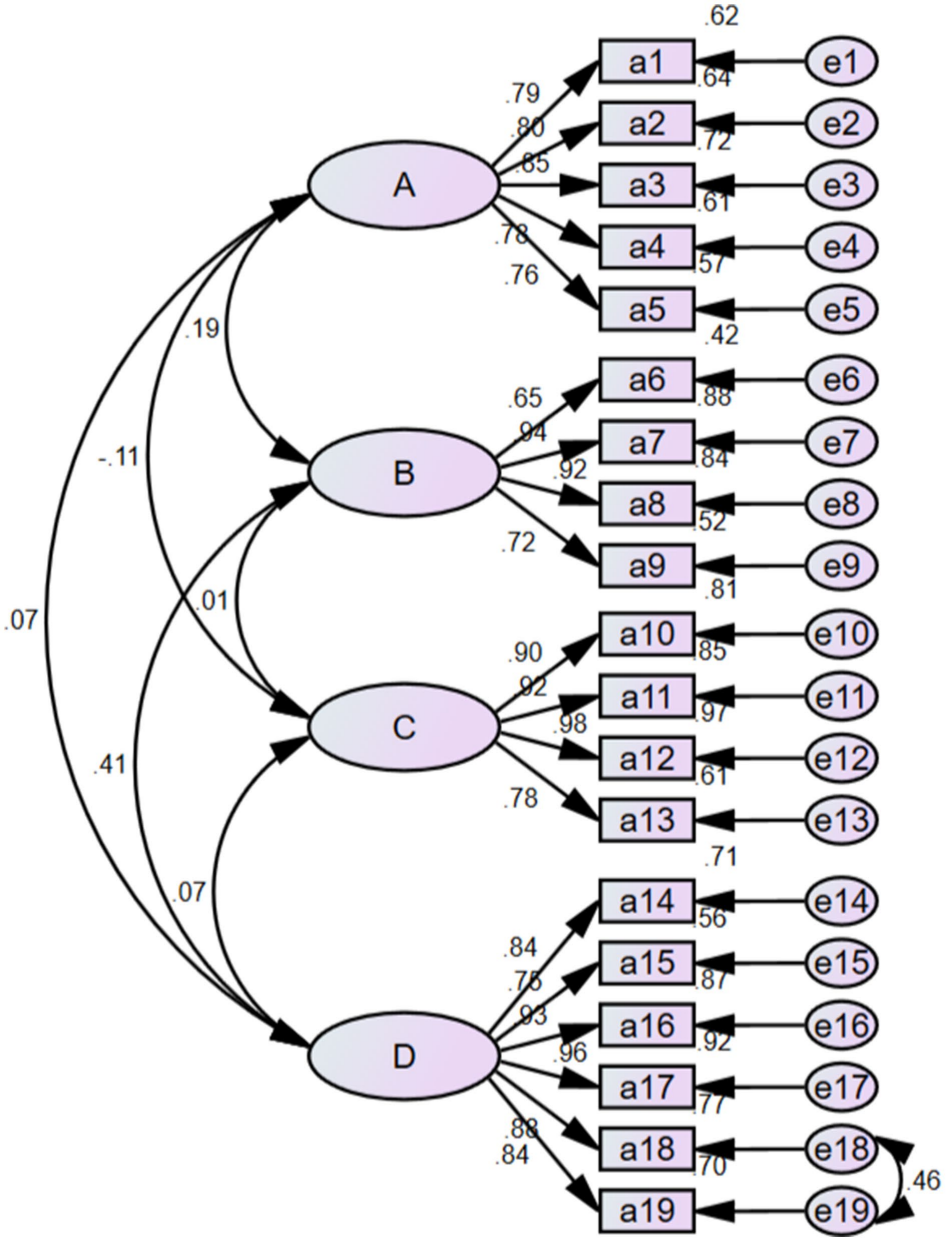
Standardized six-factor model of the Chinese version of OALS. A: preventive behavior; B: food/diet; C: physical activity; D: quality of relationships.

The purpose of confirmatory factor analysis is to validate the hypothesized relationships between items and factors. In this study, the confirmatory factor analysis was conducted using AMOS 24.0 software, employing a four-factor structure model and adhering to the maximum likelihood estimation method. Based on the modification index, the model was optimized by incorporating residual paths for e18 and e19. The results are illustrated in Figure 2. The optimized model demonstrated favorable fit indices: CMIN/DF = 1.446, RMSEA = 0.053, CFI = 0.979, GFI = 0.901, TLI = 0.976, and IFI = 0.980.

## 4 Discussion

Currently, there is a paucity of relevant research reports concerning the lifestyle of the older adult in China. One significant reason for this gap is the absence of measurement tools specifically designed to assess the lifestyle of this demographic. Lifestyle factors profoundly influence the older adult, being closely associated with their quality of life and safety (27, 28). Consequently, managing the lifestyle of the older adult and promoting active aging has emerged as a critical objective within the public health sector (29, 30). As the global economy develops and social medicine advances, the issue of global aging is becoming increasingly pressing, presenting substantial challenges for countries worldwide, particularly China, which has a substantial older adult population. To effectively evaluate the lifestyle of the older adult in China, we introduced the Brazilian OALS and conducted a psychometric assessment involving 393 older adult participants. The results indicate that the Chinese version of the OALS demonstrates strong reliability and validity. Consistent with the findings of Ferreira et al. (4), our study corroborated the four-factor structure. Notably, the higher retest reliability (0.964) may reflect a cultural emphasis on consistency among Chinese respondents. The “Quality of Relationships” dimension may possess unique connotations in China, where filial piety and intergenerational co-residence are culturally prioritized. Future studies should investigate whether these items align with local perceptions of social support. Implementing this tool in China will enhance our understanding of the lifestyle circumstances of older adults, providing a theoretical foundation for clinicians and relevant government agencies to develop targeted lifestyle interventions. Additionally, we recommend validating the scale in diverse settings (e.g., nursing homes versus community-dwelling older adult) to tailor interventions to specific populations.

Our translation work was conducted following the Brislin two-way translation method. Experts in relevant fields were invited to perform cultural adjustments to the translated scale in accordance with established guidelines and conventions of Chinese expression (16). Item analysis revealed that the Chinese version of the OALS was well differentiated among its items, with each item effectively assessing the lifestyle of the older adult. Notably, the deletion of any single item did not result in a Cronbach’s α coefficient that exceeded the reliability of the overall scale; thus, all 19 items were retained.

In this study, we employed Cronbach’s α coefficient, split-half reliability, and test–retest reliability to evaluate the reliability of the Chinese version of OALS. The Cronbach’s α coefficient for the translated scale was found to be 0.824, which is comparable to that of the original scale (4). This similarity may be attributable to the analogous social systems and cultures of China and Brazil. Furthermore, the split-half reliability and test–retest reliability of the Chinese version of OALS were 0.792 and 0.964, respectively, both of which were higher than those of the original scale (4). The high retest reliability of 0.964 may indicate that participants are familiar with the scale. To minimize the learning effect, it is advisable to enforce a two-week interval between assessments. This indicates that the Chinese version of the OALS possesses strong temporal stability. In summary, the Chinese version of OALS demonstrates robust reliability.

In this study, both content validity and structural validity were employed to evaluate the structural validity of the Chinese version of the OALS. Content validity reflects the extent to which the items on the scale align with the intended measurement objectives, while structural validity pertains to the accurate correspondence between the theoretical constructs of the scale and the actual measurements obtained (31). The content validity analysis yielded an I-CVI range of 0.857 to 1.000, with an S-CVI value of 0.955, both exceeding the reference values for content validity of 0.78 and 0.90 (32). In the structural validity analysis, exploratory factor analysis identified four factors with eigenvalues greater than 1, which accounted for 76.682% of the total variance in the data. Each factor loading was above 0.4, and the factor assignment for each item was consistent with that of the original scale, indicating that each item effectively assesses the lifestyle of the older adult (4). Furthermore, confirmatory factor analysis corroborated the proposed four-factor structure of the Chinese version of the OALS, with all fit indices demonstrating favorable results. In conclusion, the Chinese version of the OALS exhibits strong validity within the older adult population.

### 4.1 Limitations

There are some limitations to this study. First of all, although the sample size of this study is sufficient, data were only collected from the Heilongjiang, Guizhou, Jiangxi, and Anhui provinces. This limitation may result in regional biases. Future studies should encompass regions with distinct socio-economic profiles (e.g., coastal versus inland) to enhance the generalizability of the findings. Second, OALS is a self-reported scale, so reporting bias is inevitable, and the results may be influenced by societal expectations bias. In addition, further validation of OALS in different cultural and medical Settings is recommended in the future to improve the external validity of this study.

## 5 Conclusion

The Chinese version of the OALS scale consists of 19 items and comprehensively evaluates the lifestyle of the older adult across four dimensions: Preventive Behavior, Food/Diet, Physical Activity, and Quality of Relationships. This study demonstrates that the Chinese version of the OALS possesses strong psychometric properties, making it suitable for further implementation in China. This validated scale can serve as a fundamental tool for designing targeted intervention programs for older adult health management in China.

## Data Availability

The raw data supporting the conclusions of this article will be made available by the authors, without undue reservation.
